# Molecular Detection of Tick-Borne Bacterial Pathogens in Ticks and Rodents from the China–Vietnam Border

**DOI:** 10.3390/vetsci12030256

**Published:** 2025-03-10

**Authors:** Hongbo Liu, Wenwei Xiao, Xinying Du, Jingzhuang Xue, Hui Wang, Qi Wang, Yule Wang, Huiqun Jia, Hongbin Song, Shaofu Qiu

**Affiliations:** 1 Chinese PLA Center for Disease Control and Prevention, Beijing 100071, China; liuhb0115@163.com (H.L.);; 2 Guangzhou Center for Disease Control and Prevention, Guangzhou 510440, China; 3 College of Life Science and Technology, Beijing University of Chemical Technology, Beijing 100029, China

**Keywords:** China–Vietnam border, molecular detection, rodents, tick-borne bacterial pathogens, ticks

## Abstract

Ticks and tick-borne diseases constitute a crucial focus for the health of both humans and animals worldwide. Although numerous studies on tick-borne diseases have been conducted in China, reports on tick-borne pathogens in ticks and rodents from the China–Vietnam border are scarce. In this study, we investigated tick-borne bacterial pathogens, including *Rickettsia*, Anaplasmataceae, and *Borrelia*, in nine rodents (*Rattus norvegicus*) and 88 ticks collected from cattle and rodents in Jingxi, a city at the China–Vietnam border. Through molecular detection and sequence analysis, four known tick-borne pathogens were identified. Specifically, *Rickettsia japonica* was detected in 46.3% (37/80) of *Haemaphysalis cornigera*; *Anaplasma phagocytophilum* and *Candidatus* Neoehrlichia mikurensis were identified in one *Ixodes granulatus* and one rodent, respectively; and *Borrelia valaisiana* was detected in two *I. granulatus*. Additionally, a potentially novel species of *Rickettsia*, provisionally named *Rickettsia* sp. JX, was detected in 41.3% (33/80) of *Ha. cornigera*, one *Rhipicephalus microplus*, three *I. granulatus*, and nine rodents, whereas a potentially novel species of *Borrelia*, tentatively named *Borrelia* sp. JX, was detected in one *I. granulatus*. To the best of our knowledge, this is the first report on tick-borne bacterial pathogens in ticks and rodents from the China–Vietnam border. These results expand the knowledge of the geographical distribution and vector diversity of tick-borne bacterial pathogens in China and are conducive to the evaluation of thee potential public health risk.

## Simple Summary

Tick-borne bacterial pathogens are a threat to both animal and human health worldwide. In China, emerging and re-emerging tick-borne diseases have inspired investigations of ticks and tick-borne pathogens in recent years. The aim of this study was to evaluate tick-borne bacterial pathogens, including *Rickettsia*, *Anaplasma*, *Ehrlichia*, *Candidatus* Neoehrlichia, and *Borrelia*, in ticks and rodents from the China–Vietnam border, an area that has limited research on tick-borne pathogens. Four known tick-borne pathogens and two potentially novel pathogens were identified in this study. These results provide the first characterization of tick-borne bacterial pathogen diversity at the China–Vietnam border and are useful to researchers and individuals, not only from China but also from Vietnam, for the prevention of tick-borne diseases.

## 1. Introduction

Ticks are blood-sucking arthropods that parasitize vertebrates, such as livestock, wildlife, and humans, worldwide, ranking second only to mosquitoes as vectors of infectious diseases [1]. These arthropods undergo a three-stage life cycle (larva, nymph, adult), typically requiring a blood meal from different hosts at each stage. During the blood feeding process, ticks can transmit a variety of pathogens, including viruses, bacteria, and protozoa, thereby posing a significant threat to the health of both humans and animals [2,3]. The primary bacterial pathogens responsible for human diseases transmitted by ticks include *Rickettsia*, *Anaplasma*, *Ehrlichia*, *Candidatus* Neoehrlichia, and *Borrelia*. In recent years, climate warming and environmental changes have facilitated the geographic expansion of tick populations, resulting in an increase in tick-borne diseases, including emerging and re-emerging infectious diseases [4,5,6]. More than 33 emerging tick-borne pathogens have been detected in mainland China since the early 1980s [7,8,9,10,11,12,13]. However, the symptoms of tick-borne diseases (TBDs), such as fever, headache, fatigue, and myalgia, are analogous to those of influenza or the common cold, which may initially lead to misdiagnosis and delayed treatment [14,15,16].

*Rickettsia* species belong to the genus *Rickettsia*, which includes more than 70 species (including 30 candidate species). They have been categorized into four groups, the spotted fever group (SFG), the typhus group (TG), the ancestral group (AG), and the transitional group, on the basis of their phenotypic traits, encompassing ecological and epidemiological features, clinical information, and mouse serotyping results [17,18]. SFG *rickettsiae* (SFGR) are predominantly maintained and transmitted by ticks. In China, SFGR were mainly detected in *Dermacentor silvarum*, *D. nuttalli*, *Ixodes persulcatus*, *Haemaphysalis longicornis*, *Rhipicephalus microplus*, and *Hyalomma asiaticum* [11,19]. A previous study in Southwestern China showed an SFGR infection rate of 36.1% in ticks [20].

*Anaplasma*, *Ehrlichia*, and *Candidatus* Neoehrlichia are tick-borne bacterial pathogens in the family Anaplasmataceae. The genus *Anaplasma* comprises eight *Anaplasma* species, including *A. phagocytophilum*, *A. ovis*, *A. bovis*, *A. marginale*, *A. platys*, *A. centrale*, and two newly discovered species, *A. odocoilei* and *A. capra* [21]. *A. capra*, an emerging tick-borne pathogen, was initially detected in humans, goats, and *I. persulcatus* ticks in Northeastern China [22]. Moreover, *A. phagocytophilum*, which is the most frequently documented agent in Anaplasmataceae in China, was first detected in *I. persulcatus* from Heilongjiang Province in 1997, with a minimum infection rate of 0.8% [11,19]. The genus *Ehrlichia* includes six validated species, *E. chaffeensis*, *E. muris*, *E. ewingii*, *E. ruminantium*, *E. canis*, and *E. minasensis*, all of which have been detected in China [19,23,24,25]. The genus *Candidatus* Neoehrlichia is a new cluster in Anaplasmataceae. Until recently, six species were known in this genus, namely *Candidatus* N. mikurensis, *Candidatus* N. lotoris, *Candidatus* N. australis, *Candidatus* N. australis, *Candidatus* N. Tanzania, and *Candidatus* N. chilensis [26,27]. *Candidatus* N. mikurensis has been detected in *D. silvarum*, *Ha. Concinna*, *Ha. longicornis*, and *I. persulcatus* in China, with an infection rate of 1.6% reported in a prior study from Northeastern China [28].

*Borrelia burgdorferi* sensu lato complexes, which belong to the genus *Borrelia*, are the etiological agents of Lyme borreliosis. Although there are more than 20 genospecies in the *B. burgdorferi* sensu lato complexes, *B. garinii*, *B. afzelii*, and *B. burgdorferi* sensu stricto are the predominant pathogens responsible for Lyme borreliosis [19,29]. In China, ticks known to be vectors of *Borrelia burgdorferi* include *I. granulatus*, *Hy. asiaticum*, *I. persulcatus*, and *Ha. concinna*, with *I. granulatus* exhibiting the highest prevalence at 24% [29].

The China–Vietnam border is located in Southwestern China, which is mountainous and abundant in biological resources, offering an ecological and biological foundation for the survival and reproduction of ticks, rodents, and tick-borne pathogens. Notably, the ecological continuity of the China–Vietnam border creates shared habitats for ticks and hosts. In Northern Vietnam, pathogens such as *Rickettsia* spp., *Anaplasma platys*, and *Ehrlichia canis* have been detected in ticks and dogs [30,31]. However, at present, information regarding tick-borne pathogens in this region is limited. The objective of this study was to determine the existence and molecular characteristics of tick-borne bacterial pathogens in ticks and rodents from the China–Vietnam border.

## 2. Materials and Methods

### 2.1. Sample Collection and Species Identification

**Figure 1:**
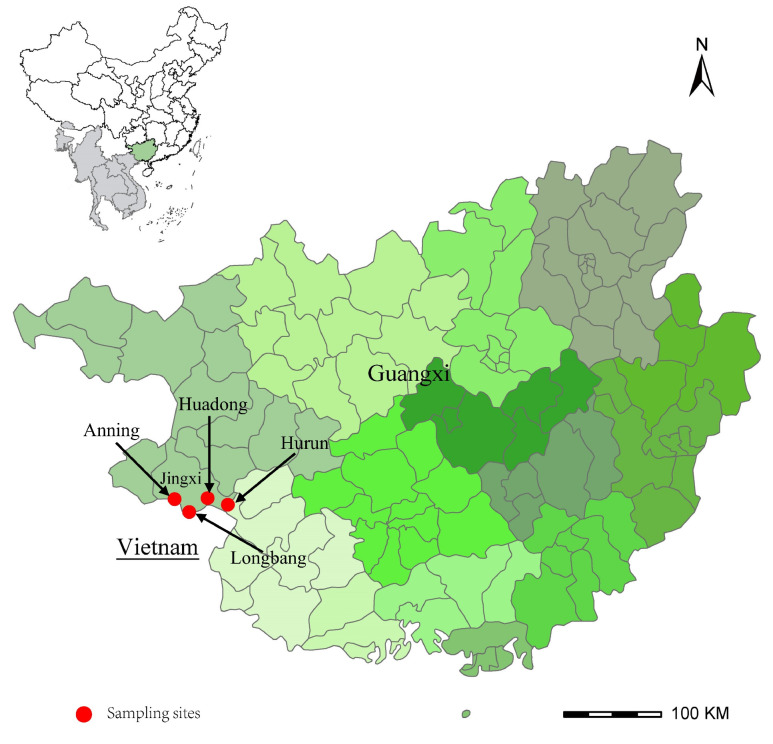
Map of the sampling sites in this study. The red dots are sampling sites in Jingxi City, Guangxi Province.

The study area and sample sites were located in Jingxi City, Guangxi Province, Southwestern China (Figure 1). Jingxi is mainly characterized by karst plateau landforms at the border between China and Vietnam. In May 2020, ticks were initially collected from the body surfaces of cattle. To investigate tick-borne pathogens in rodents, rodents were captured using peanut bait, and ticks were collected from each rodent. All feeding ticks were removed from the hosts using steel forceps and placed in individual 5 mL tubes, each of which was hermetically sealed with breathable cotton. A total of 88 ticks and nine rodents were collected from four villages. All ticks were parasitic, with 85 specimens collected from 10 cattle and the remaining three obtained from a single rodent. The collected ticks were identified morphologically under a stereoscopic microscope according to existing morphological criteria and by molecular methods using the 16S ribosomal RNA and mitochondrial cytochrome c oxidase I genes, as previously described [32,33]. Spleen specimens were removed from each rodent after the identification of the species by morphology. All samples were stored at −80 °C to ensure their quality for further analysis.

### 2.2. DNA Extraction

Before DNA extraction, each tick was surface-sterilized in 75% ethanol and then rinsed twice with phosphate-buffered saline (PBS) to remove environmental contaminants. Individual ticks and 300 mg of each rodent tissue sample were singly homogenized in a 1.5 mL centrifuge tube with 2 mm stainless steel beads via an automated tissue homogenizer (TGrinder H24, Tiangen Biotech, Beijing, China). DNA extraction was performed on 200 µL of each homogenate using a DNeasy tissue kit (QIAGEN, Hilden, Germany), according to the manufacturer’s instructions.

### 2.3. Molecular Detection of Tick-Borne Bacterial Pathogens

The molecular identification of tick-borne bacterial pathogens was carried out using PCR assays. All samples of ticks and rodents were screened for spotted fever group *Rickettsiae* (SFGR), Anaplasmataceae, and *Borrelia*. The primers used in this study are listed in Table 1. Primer pair Rr190.70f/Rr190.602r, targeting the outer membrane protein A gene (*ompA*), and semi-nested primer pairs CS2d/CSEndr, RpCS877f/CSEndr, and CS2d/RpCS1258r, targeting the citrate synthase gene (*gltA*), were used for screened SFGR [34]. Semi-nested primer pairs Eh-out1/Eh-3-17, Eh-out1/Eh-out2, and Eh-out2f/Eh-3-17, targeting the 16S rRNA gene, were used for the detection of Anaplasmataceae and SFGR [35]. For the identification of *Borrelia* spp., conventional PCRs targeting the 16S rRNA gene and the flagellin gene (*fla*) were performed using the primer pairs 16S1/16S2 and FlaF/FlaR, respectively [36,37]. A negative control (distilled water) was included in each amplification. The PCR products were visualized on a 1.0% agarose gel stained with ethidium bromide under UV light.

### 2.4. Sequencing and Phylogenetic Analysis

PCR-positive products were purified using the EasyPure Quick Gel Extraction Kit (TransGen Biotech, Beijing, China) and sequenced using a commercial sequencing service (Tianyi Huiyuan, Beijing, China). The obtained nucleotide sequences were compared with those available in GenBank using nucleotide BLAST (https://blast.ncbi.nlm.nih.gov, accessed on 1 March 2025) (National Center for Biotechnology Information, NCBI, Bethesda, MD, USA), and multiple sequence alignment was performed using the ClustalW multiple alignment tool with the default parameters in MEGA 7.0 [38]. Phylogenetic trees were constructed via MEGA 7.0 based on the maximum likelihood method with the Kimura two-parameter method, and a bootstrap analysis was performed with 1000 replicates [39]

## 3. Results

### 3.1. Sample Collection

A total of 88 ticks were collected during the study. Morphological and molecular identification revealed that the ticks belonged to three species: 80 (90.9%) to *Haemaphysalis cornigera*, five (5.7%) to *Rhipicephalus microplus*, and three (3.4%) to *Ixodes granulatus* (Table 2). *Ha. cornigera* and *R. microplus* were collected from cattle, whereas *I. granulatus* was gathered from one of the nine rodents, all of which were identified as *Rattus norvegicus* (*Rat. norvegicus*). Specifically, 73 *Ha. cornigera* were obtained from Anning Village, along with three *R. microplus* from Longbang Village. Furthermore, two *R. microplus* and seven *Ha. cornigera* were obtained from Hurun Village, while Huadong Village yielded three *I. granulatus* and nine *Rat. norvegicus*.

### 3.2. Detection and Characterization of SFGR

Rickettsial DNA was detected in 74 ticks and nine *Rat. norvegicus* through the application of *ompA* gene primers, yielding an overall infection prevalence of 84.1% in ticks and 100% in *Rat. norvegicus*. Furthermore, the *gltA* and 16S rRNA genes were amplified and sequenced for all positive samples. Two SFGR species, namely *R. japonica* and a genetic variant of *Rickettsia* (tentatively designated as *Rickettsia* sp. JX), were identified through the analysis of partial fragments of the *gltA*, *ompA*, and 16S rRNA genes. Specifically, *R. japonica* was detected in 37 (46.3%) *Ha. cornigera* collected from cattle. *Rickettsia* sp. JX was identified in nine (100%) *Rat. norvegicus* and 37 (42.1%) ticks, including 33 (41.3%) *Ha. cornigera*, one *R. microplus* sampled from cattle, and three *I. granulatus* collected from *Rat. norvegicus* (Table 2).

**Figure 2:**
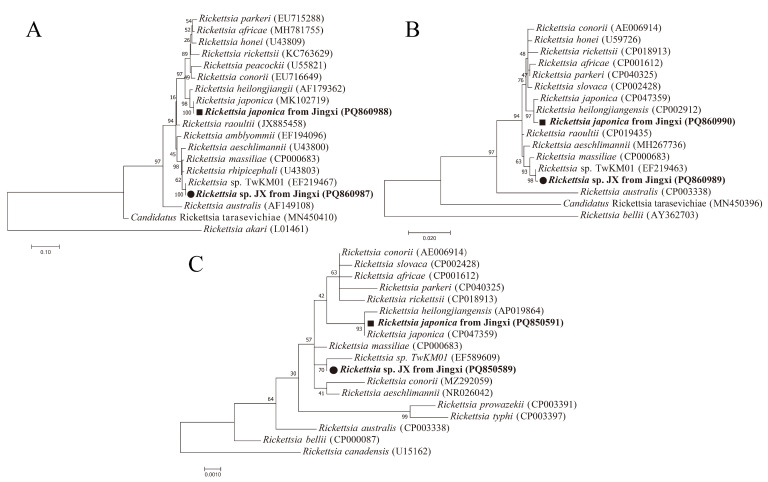
Phylogenetic trees of genus *Rickettsia* constructed using the maximum likelihood method in MEGA 7.0. (**A**) Phylogenetic tree based on *ompA* genes (500 bp). (**B**) Phylogenetic tree based on *gltA* genes (1099 bp). (**C**) Phylogenetic tree based on 16S rRNA genes (1397 bp). Scale bars indicate estimated evolutionary distances. The values at the nodes represent the percentage support from 1000 bootstrap replicates. Circles and squares indicate *Rickettsia* sp. JX and *Rickettsia japonica* identified in this study, respectively.

The sequences of *R. japonica* derived from 37 *Ha. cornigera* were found to be identical across all three amplified genes. The determined *ompA* gene sequences showed 100% similarity to *R. japonica* strain LA16/2015 (CP047359) isolated from a human in Zhejiang Province, China, whereas the *gltA* and 16S rRNA gene sequences were 99.5% and 99.9% identical to the corresponding sequences of *R. japonica* strain LA16/2015, respectively. Phylogenetic analyses based on the above-mentioned three rickettsial genes revealed that the obtained *R. japonica* from Jingxi was clustered with the previous *R. japonica* strain LA16/2015, differing from other SFGR (Figure 2).

The sequences of *Rickettsia* sp. JX generated from 33 *Ha. cornigera*, one *R. microplus*, three *I. granulatus*, and nine *Rat. norvegicus* were identical to each other for each gene. The sequence analysis by BLAST showed that the sequences were closest to an unvalidated species, *Rickettsia* sp. TwKM01 (EF589609), with 100%, 99.7%, and 99.8% identity for the *ompA*, *gltA*, and 16S rRNA genes, respectively. However, the *ompA* sequence was 98% identical to the corresponding sequence of *R. massiliae* (CP000683) and 97.4% identical to *R. aeschlimannii* (U43800). The *gltA* sequence showed 99.4% similarity to *R. massiliae* (CP000683) and 97.4% similarity to *R. aeschlimannii* (MH267736). The 16S rRNA sequence shared 99.7% identity with *R. massiliae* (CP000683) and 99.5% identity with *R. aeschlimannii* (NR026042). The phylogenetic analyses suggested that *Rickettsia* sp. JX was closely related to *Rickettsia* sp. TwKM01, while it was distinct from other validated *Rickettsia* species and formed a separate clade.

### 3.3. Detection and Characterization of Anaplasmataceae

**Figure 3:**
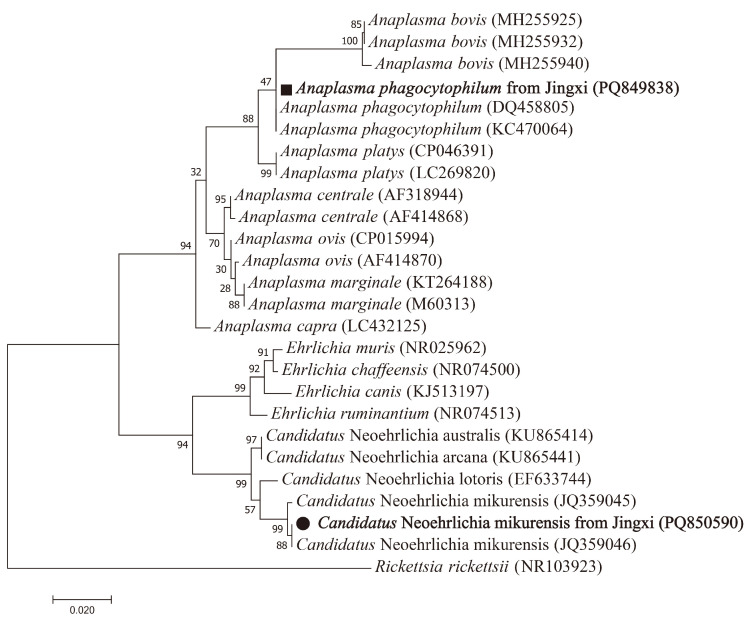
Phylogenetic tree based on 16S rRNA genes (735 bp) of Anaplasmataceae using the maximum likelihood method in MEGA 7.0. Scale bars indicate estimated evolutionary distances. The values at the nodes represent the percentage support from 1000 bootstrap replicates. Circles and squares indicate *Candidatus* N. mikurensis and *A. phagocytophilum* identified in this study, respectively.

In the investigation of Anaplasmataceae, one *I. granulatus* and one *Rat. norvegicus* were positive for the 16S rRNA gene. Through the analysis of the 16S rRNA gene, *A. phagocytophilum* and *Candidatus* N. mikurensis were identified in the above-mentioned *I. granulatus* and *Rat. norvegicus*, respectively. The sequence of *A. phagocytophilum* in this study was 100% identical to that of *A. phagocytophilum* from Zhejiang (DQ458805). The sequence of *Candidatus* N. mikurensis from Jingxi displayed 99.5% similarity to that of *Candidatus* N. mikurensis from Zhejiang (JQ359046) (Figure 3).

### 3.4. Detection and Characterization of Borrelia spp.

For *Borrelia* spp., all three *I. granulatus* obtained from *Rat. norvegicus* were positive for the 16S rRNA and *fla* genes. Two *Borrelia* species, namely *B. valaisiana* and a genetic variant of *Borrelia* (provisionally named *Borrelia* sp. JX), were determined through the analysis of the sequences of the 16S rRNA and *fla* genes.

**Figure 4:**
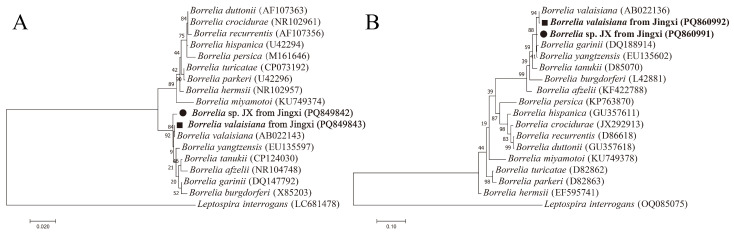
Phylogenetic trees of genus *Borrelia* constructed using the maximum likelihood method in MEGA 7.0. (**A**) Phylogenetic tree based on 16S rRNA genes (1381 bp). (**B**) Phylogenetic tree based on *fla* genes (560 bp). Scale bars indicate estimated evolutionary distances. The values at the nodes represent the percentage support from 1000 bootstrap replicates. Circles and squares indicate *Borrelia* sp. JX and *B. valaisiana* identified in this study, respectively.

The sequences of *B. valaisiana* obtained from two *I. granulatus* shared 100% identity with each other and had 99.9% and 99.8% identity in the 16S rRNA and *fla* genes, respectively, to the *B. valaisiana* detected in Zhejiang (AB022143, AB022136) (Figure 4).

The 16S rRNA gene sequence of *Borrelia* sp. JX from one *I. granulatus* was 99.5% and 99.1% identical to the corresponding sequences of *B. valaisiana* detected in Zhejiang (AB022143) and *B. yangtzensis* identified in Guizhou (EU135597), respectively. The *fla* gene sequence of *Borrelia* sp. JX showed 98.6% and 98.2% similarity to *B. valaisiana* discovered in Zhejiang (AB022136) and *B. yangtzensis* identified in Guizhou (EU135602), respectively. The phylogenetic analyses based on the 16S rRNA and *fla* genes indicated that *Borrelia* sp. JX was different from other validated *Borrelia* species and formed a separate clade.

### 3.5. Co-Infection in Individual Ticks and Rodents

The co-infection of *Rickettsia* sp. JX, *A. phagocytophilum*, and *B. valaisiana* was observed from one *I. granulatus*, while one *Rat. norvegicus* was co-infected by *Rickettsia* sp. JX and *Candidatus* N. mikurensis.

## 4. Discussion

Guangxi Province is located in the southwestern part of China, with a complex geographic environment, and possesses a border with Vietnam, extending over 1000 km. Due to the high forest cover, high species diversity, and frequent trade along the China–Vietnam border, the diversity of tick-borne pathogens deserves attention. In this study, we investigated the presence of *Rickettsia*, Anaplasmataceae, and *Borrelia* species in ticks and rodents collected from Jingxi, a city located at the China–Vietnam border. Through molecular detection and sequence analysis, two species of SFGR, specifically *R. japonica* and *Rickettsia* sp. JX, as well as *A. phagocytophilum*, *Candidatus* N. mikurensis, and two species of *Borrelia*, namely *B. valaisiana* and *Borrelia* sp. JX, were identified. Additionally, 80 (90.9%) ticks in this study were identified as *Ha. cornigera*, which has received limited study in China.

*Rickettsia japonica* was initially discovered in Japan in 1984 and has the capacity to cause Japanese spotted fever in humans, presenting with fever, headache, rash, eschar, and malaise [40]. Japanese spotted fever is predominant in Japan; however, cases have also been reported in China, South Korea, and Thailand [41,42,43]. In China, *R. japonica* has been detected in *H. longicornis*, *H. hystricis*, *H. flava*, *H. taiwana*, and *R. microplus* from the central, southeastern, and northeastern regions [19]. We identified *R. japonica* in *Ha. cornigera* from Jingxi. Thus, this study provides the first evidence of *R. japonica* in *Ha. cornigera* in Southwestern China. In accordance with the genetic criteria for the definition of novel rickettsial species, an isolate can be categorized as a potential new *Rickettsia* species if it shows no more than one of the following levels of nucleotide similarity with a validated *Rickettsia* species: ≥99.8%, ≥99.9%, and ≥98.8% for the 16S rRNA, *gltA*, and *ompA* genes, respectively [44]. Therefore, we regarded the newly detected *Rickettsia* sp. JX as a potential novel SFGR species. *Rickettsia* sp. JX recovered from Jingxi was closest in identity to *Rickettsia* sp. TwKM01, which was identified in *R. haemaphysaloides* from Taiwan Province, China [45]. However, in this study, *Rickettsia* sp. JX was detected in *Rat. norvegicus* and three species of ticks collected from Jingxi, including *Ha. cornigera*, *R. microplus*, and *I. granulatus*, which suggests that *Rickettsia* sp. JX is a major tick-borne pathogen prevalent in this region.

*Anaplasma phagocytophilum* is widely distributed globally, featuring a broad range of mammalian hosts and high genetic diversity. Moreover, six genera of ticks composed of 22 species serve as vectors of *A. phagocytophilum* in China [19]. In this study, *A. phagocytophilum* was confirmed in one *I. granulatus* with the 16S rRNA gene. *Candidatus* N. mikurensis has been detected mainly in *Dermacentor silvarum*, *H. longicornis*, and various rodents in China [11]. A previous study on rodents indicated that *Rat. andamanensis* and *Rat. losea* were the hosts of *Candidatus* N. mikurensis in Guangxi [46]. Additionally, in this study, *Candidatus* N. mikurensis was detected in *Rat. Norvegicus* in Guangxi, thereby expanding its host range in this region. Importantly, the findings from the present study align with an independent report of *Candidatus* N. mikurensis in *Rat. norvegicus* from Henan Province in Central China [47], collectively expanding the known host range and geographical distribution of this pathogen across distinct ecological regions.

Lyme borreliosis was first reported in America in 1975 and subsequently identified in Heilongjiang Province, China in 1986 [48]. Until recently, a total of nine genospecies of *B. burgdorferi* have been detected in China, among which *B. garinii* was the most predominant genospecies, followed by *B. afzelii* and *B. valaisiana*. In terms of the transmission of Lyme disease, *I. granulatus*, *Hy. asiaticum*, *I. persulcatus*, and *H. concinna* play significant roles in China [29]. Here, we identified *B. valaisiana* in two *I. granulatus* collected from *Rat. Norvegicus*. In China, *B. valaisiana* was initially detected in Zhejiang and was the prevalent genospecies in Southwestern China [36]. Additionally, a genetically related but distinct genospecies of *B. valaisiana*, namely the *B. valaisiana*-related genospecies, has been discovered in many areas of China [37,49,50]. Based on the 16S rRNA and *fla* genes, we detected a novel *Borrelia* sp. JX in one *I. granulatus*, belonging to the *B. valaisiana*-related genospecies. Although rodents are the major hosts of *Borrelia* species and the three *Borrelia*-positive ticks were gathered from rodents, no *Borrelia* species were detected in the nine *Rat. norvegicus* in the present study.

All *Ha. cornigera* and *R. microplus* with a high positivity rate for *Rickettsia* were collected from cattle; however, we did not obtain any blood samples from the cattle, which is one of the limitations of the study. Another limitation is that only the 16S rRNA gene was acquired for *A. phagocytophilum* and *Candidatus* N. mikurensis, affecting our comprehension of their genetic traits.

## 5. Conclusions

To our knowledge, this is the first study to investigate various tick-borne bacterial pathogens in ticks and rodents collected from the China–Vietnam border. This study provides information on the presence of *R. japonica*, *A. phagocytophilum*, *Candidatus* N. mikurensis, and *B. valaisiana* in the study region, which will promote the epidemiological and molecular understanding of tick-borne bacterial pathogens at the China–Vietnam border. Additionally, two potentially novel pathogens, namely *Rickettsia* sp. JX and *Borrelia* sp. JX, were identified for the first time in this region. These findings provide valuable insights for veterinarians and public health specialists to effectively control tick-borne diseases in this region. Although the sample size was not large, six species of tick-borne bacterial pathogens and co-infections were detected, indicating that the variety of tick-borne pathogens in this region is considerable and merits further investigation.

## Figures and Tables

**Table 1:** Primers used in this study.

Pathogens	Gene	Primer Name	Expected Size (bp)	Sequence (5′-3′)	Reference
SFGR	*ompA*	Rr190.70f	532	ATGGCGAATATTTCTCCAAAA	
		Rr190.602r		AGTGCAGCATTCGCTCCCCCT	
	*gltA*	CS2d	1256	ATGACCAATGAAAATAATAAT	[34]
		RpCS1258r		ATTGCAAAAAGTACAGTGAACA	
		CSEndr	750	CTTATACTCTCTATGTACA	
		RpCS877f		GGGGACCTGCTCACGGCGG	
SFGR and Anaplasmataceae	16S rRNA	Eh-out1	1438	TTGAGAGTTTGATCCTGGCTCAGAACG	
		Eh-3-17		GATAGCGGAATTCCTAGTGTAGAGGTG	
		Eh-out1	660	TTGAGAGTTTGATCCTGGCTCAGAACG	[35]
		Eh-out2		TAAGGTGGTAATCCAGC	
		Eh-out2f	890	CACCTCTACACTAGGAATTCCGCTATC	
		Eh-3-17		GATAGCGGAATTCCTAGTGTAGAGGTG	
*Borrelia*. spp.	16S rRNA	16S1	1523	ATAACGAAGAGTTTGATCCTGGC	
		16S2		CAGCCGCACTTTCCAGTACG	[36]
	*fla*	FlaF	588	TTAGGTTTTCAATAGCATACTCAG	
		FlaR		GCAGTTCAATCAGGTAACGG	[37]

**Table 2:** The SFGR detected in ticks collected from the China–Vietnam border.

Tick	Host	No. of Ticks (%)	Number of SFGR (%, 95% CI)		
			*Rickettsia japonica*	*Rickettsia* sp. JX	Total
*Ha. cornigera*	cattle	80 (90.9)	37 (46.3, 35.2–57.7)	33 (41.3, 30.5–52.8)	70 (87.5, 77.8–93.5)
*R. microplus*	cattle	5 (5.7)	0	1 (20, 1.1–70.1)	1 (20, 1.1–70.1)
*I. granulatus*	*R. norvegicus*	3 (3.4)	0	3 (100, 31–100)	3 (100, 31–100)
Total		88	37 (42.1, 31.8–53.1)	37 (42.1, 31.8–53.1)	74 (84.1, 74.4–90.7)

## Data Availability

All relevant data are provided in the manuscript.
